# Heterogeneity in Risk of Pelvic Inflammatory Diseases After Chlamydia Infection: A Population-Based Study in Manitoba, Canada

**DOI:** 10.1093/infdis/jiu483

**Published:** 2014-12-01

**Authors:** Bethan Davies, Helen Ward, Stella Leung, Katy M. E. Turner, Geoff P. Garnett, James F. Blanchard, B. Nancy Yu

**Affiliations:** 1 Department of Infectious Disease Epidemiology, School of Public Health, Imperial College London; 2 School of Social and Community Medicine and School of Clinical Veterinary Science, University of Bristol, United Kingdom; 3 Centre for Global Public Health, Department of Community Health Sciences, University of Manitoba; 4 Public Health Branch, Manitoba Health, Winnipeg, Canada; 5 Bill and Melinda Gates Foundation, Seattle, Washington

**Keywords:** *Chlamydia trachomatis*, cohort study, pelvic inflammatory disease, retrospective study, epidemiology, mathematical models, cost effectiveness

## Abstract

***Background.*** The association between chlamydia infection and pelvic inflammatory disease (PID) is a key parameter for models evaluating the impact of chlamydia control programs. We quantified this association using a retrospective population-based cohort.

***Methods.*** We used administrative health data sets to construct a retrospective population-based cohort of women and girls aged 12–24 years who were resident in Manitoba, Canada, between 1992 and 1996. We performed survival analysis on a subcohort of individuals who were tested for chlamydia to estimate the risk of PID diagnosed in a primary care, outpatient, or inpatient setting after ≥1 positive chlamydia test.

***Results.*** A total of 73 883 individuals contributed 625 621 person years of follow-up. Those with a diagnosis of chlamydia had an increased risk of PID over their reproductive lifetime compared with those who tested negative (adjusted hazard ratio [AHR], 1.55; 95% confidence interval [CI], 1.43–1.70). This risk increased with each subsequent infection: the AHR was 1.17 for first reinfection (95% CI, 1.06–1.30) and 1.35 for the second (95% CI, 1.04–1.75). The increased risk of PID from reinfection was highest in younger individuals (AHR, 4.55 (95% CI, 3.59–5.78) in individuals aged 12–15 years at the time of their second reinfection, compared with individuals older than 30 years).

***Conclusions.*** There is heterogeneity in the risk of PID after a chlamydia infection. Describing the progression to PID in mathematical models as an average rate may be an oversimplification; more accurate estimates of the cost-effectiveness of screening may be obtained by using an individual-based measure of risk. Health inequalities may be reduced by targeting health promotion interventions at sexually active girls younger than 16 years and those with a history of chlamydia.

The control of *Chlamydia trachomatis* (chlamydia) is a public health priority in many high-income settings [1]. Interventions aim to prevent cases of pelvic inflammatory disease (PID) in the short term, and ectopic pregnancy and tubal infertility, in the long term [2]. Policy decisions, including the level of investment and design of chlamydia control interventions, are increasingly informed by mathematical models. These models seek to accurately describe the natural history of chlamydia and predict the impact of control interventions. The sensitivity analyses that accompany these models have consistently demonstrated that their predictions are highly influenced by the parameter used to describe the risk of progression from chlamydia to PID [3–10].

A systematic review has shown that this parameter is predominantly included as a fixed proportion in the static component of economic analyses, with an average value of 22% [11]. There are fewer examples of it being included in dynamic transmission models, but where this occurs it is again as a fixed value, with the majority falling between 20% and 30% [11]. There is not a consensus for the risk of PID after chlamydia [12], and a risk of progression of about 20% is more similar to estimates from studies of high-risk women [13, 14] than estimates from the general population, where current control interventions are applied. For example, the Prevention of Pelvic Infection randomized controlled trial set in higher-education settings found that 9.5% (95% confidence interval [CI], 4.7–18.3%) of women in the control arm who had chlamydia at baseline progressed to PID within a year [15] and a follow-up study of women tested during a preemployment health check found that PID developed within 1 year in 0% (95% CI, 0%–9.5%) [12, 16].

Population-based cohorts have demonstrated that women who test positive for chlamydia have an increased risk of PID throughout their reproductive lifetime compared with women who test negative [17, 18]. Findings of cohort studies have also suggested that the risk of PID may increase after repeated infections, although this has not yet been well characterized in a contemporary general population setting [12, 19, 20]. To explore the complex relationship between chlamydia, chlamydia testing, and PID we have constructed a retrospective population-based cohort in Manitoba, Canada, where there has been an established chlamydia control program for >2 decades. We describe the use of this cohort to estimate the risk of PID after ≥1 positive chlamydia test in women who have been tested for chlamydia.

## METHODS

The Province of Manitoba in Canada provides universal healthcare to its residents through an insurance system funded by Manitoba Health (MH), the provincial government health department, which has collected and maintained electronic data sets of all its healthcare activity since 1971 [21]. This data includes a register of persons eligible to receive healthcare, hospital inpatient and outpatient services, and physician care outside a hospital setting. By linking the information held in these data sets it is possible to create a longitudinal record of an individual's interaction with the province's healthcare system [22, 23]. This linkage is performed using a unique 9-digit personal health information number (PHIN) that is assigned to an individual at birth or when he or she registers with the insurance system (for research purposes this PHIN is provided in a “scrambled” form to pseudo-anonymize the data).

We used the MH Insurance Registry (for a description of the data set see Supplementary Online Material 1) to generate a retrospective population-based cohort of females resident in Manitoba, aged 12–24 years between 1992 and 1996, the Manitoba Women's Reproductive and Sexual Health Cohort. Women (or girls) enter this cohort on their 12th birthday, 1 January 1992, or the date they enrolled with the insurance system (latest event) and remain in the cohort until their 41st birthday, 31 December 2008, or the date they leave the insurance system (earliest event). We generated a list of PHINs for the individuals in the cohort. This was linked to the Cadham Provincial Laboratory (CPL) data set to extract records of all their chlamydia tests performed between 01 January 1992 and 31 December 2008. Then it was linked to the Medical Claims and Hospital Separations data sets to extract all their records with a diagnosis code for PID, pregnancy, or infertility from 1987 or their 12th birthday (latest event) until 31 December 2008. We then removed the duplicate records that occurred if the same event was recorded in the Medical Claims and Hospital Separations data sets.

A chlamydia test was defined as any nonserological test for chlamydia performed ≥60 days after a previous test [24]. During the study period, chlamydia was diagnosed using the Chlamydiazyme test (Abbott Laboratory) (1992–1998), the PACE 2 nucleic acid probe test (GenProbe) for urethral and cervical specimens and AMP-CT nucleic acid amplification test (NAAT) (GenProbe) for urine samples (1999–2007), and Aptima NAAT (GenProbe) (2007–2008). The International Classification of Disease (ICD) codes used to define the outcomes are presented in Supplemental Online Material 2.

A subset of the cohort was used in this analysis. We excluded individuals who (1) did not have a chlamydia test during the cohort or (2) had a diagnosis of PID, pregnancy, or infertility between 1987 or their 12th birthday (latest event) and their first test in the cohort. We limited chlamydia test records to the first 3 tests per individual and moved the date of the test backward by 1 day if it occurred on the same date as a PID diagnosis. Women (or girls) entered this cohort on the date of their first chlamydia test and were censored on their 41st birthday, 31 December 2008, the date of leaving Manitoba, or the date of their first PID diagnosis (earliest event).

We determined the incidence of chlamydia at the first, second, and third chlamydia tests and calculated the cumulative incidence of PID by the end of follow-up after each test respectively. We converted the data set into a format suitable for survival techniques. Individuals enter and exit this data set at appropriate times, so the population denominator varies daily. We used this data set to calculate the rate of PID after the first, second, and third chlamydia tests, to construct Kaplan–Meier plots of time to PID and to perform Cox proportional hazards regression to estimate the risk of PID after a positive chlamydia test adjusted for previous positive chlamydia tests during the cohort, age at test, year of test, region of residence (at time of cohort entry or within 5 years of this date if not available at time of entry; n = 232).

Institutional approval for use of the administrative healthcare data was obtained from the Health Information Privacy Committee (HIPC) at MH (project HIPC 2010/2011-48). Ethical approval for the use of pseudo-anonymized administrative health data was obtained from the Health Research Ethics Board at the University of Manitoba (Health Research Ethics Board reference H2010-373). All data were provided and hosted by the Manitoba Centre for Health Policy within the University of Manitoba. Statistical analysis was completed using SAS 9.2 software (SAS Institute).

## RESULTS

**Figure 1.**
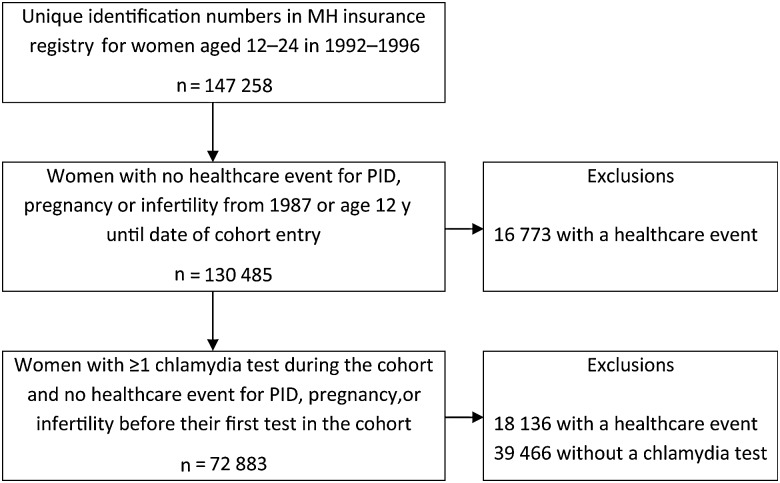
Cohort formation. Abbreviations: MH, Manitoba Health; PID, pelvic inflammatory disease.

The cohort contains 147 258 women and girls aged 12–24 years between 1992 and 1996; 72 883 (49.5%) were eligible for this analysis because they had been tested for chlamydia and did not have a recorded diagnosis of PID, pregnancy, or infertility before the date of their first chlamydia test in the cohort (Figure 1). These individuals contributed 625 621 person-years of follow-up before their 41st birthday, 31 December 2008, leaving Manitoba, or their first diagnosis of PID.

The mean age at the first chlamydia test in the cohort (equal to the age at cohort entry) was 20.5 years (SD, 4.1 years), and the mean number of chlamydia tests was 4.7 (SD, 4.0; median, 4); 58 473 individuals (80.2%) had ≥2 chlamydia tests, and 45 924 (63.0%) had ≥3 tests before censoring. The incidence of chlamydia was 5.5% at the first test, 4.6% at the second, and 4.3% at the third. The cumulative incidence of PID by the end of follow-up was 8.3% (n = 6014) after the first test in the overall cohort, 7.9% in those who tested negative, and 14.8% in those who tested positive. The cumulative incidences of PID after the second test were 7.7% overall, 7.3% negative, and 14.8% positive and 7.3%, 7.0%, and 12.7%, respectively, after the third test.

**Figure 2.**
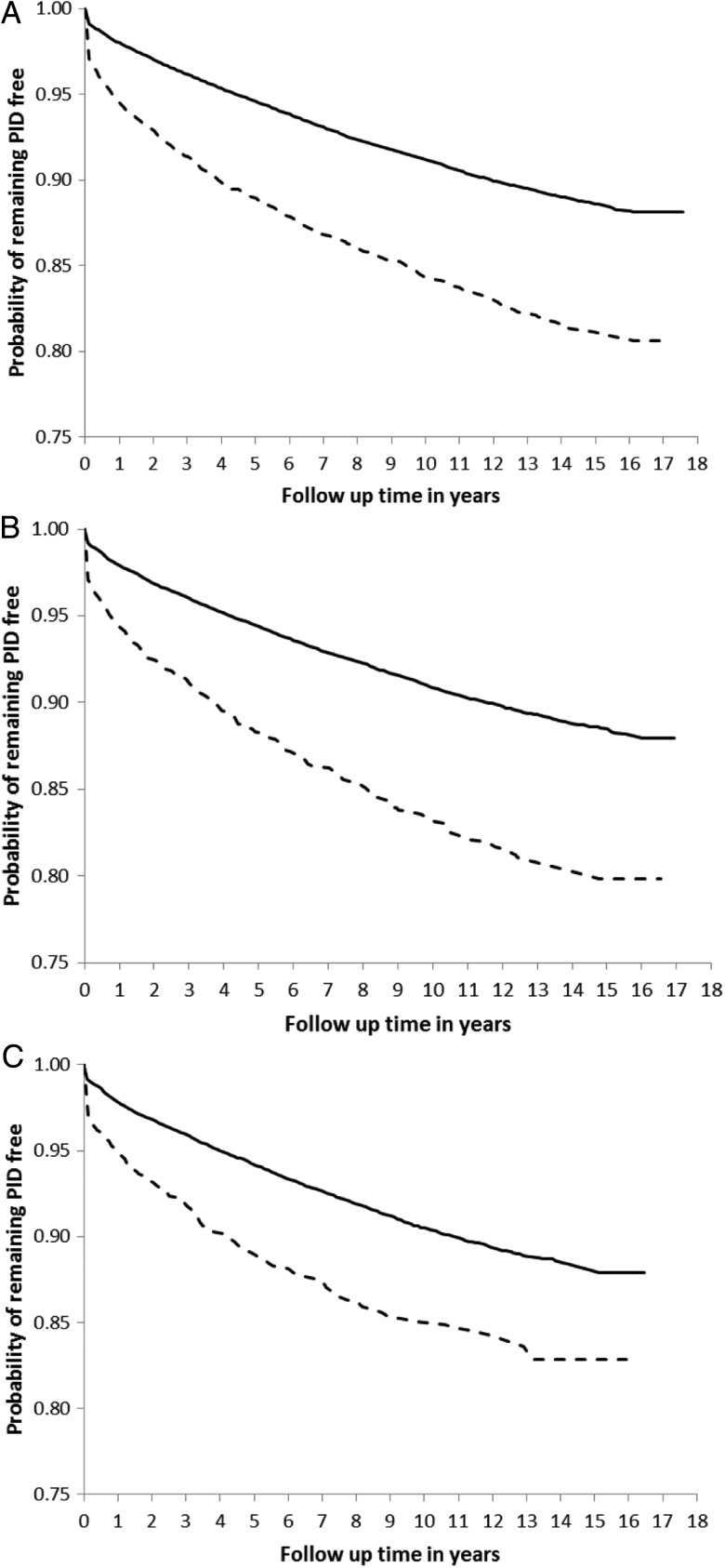
Kaplan–Meier survival curves of time to pelvic inflammatory disease (PID) by test result. After the first (*A*), second (*B*), and third (*C*) chlamydia tests. Solid = negative women; dashed = positive women.

PID occurred at a rate of 0.96, 0.80, and 0.70 per 100 person-years after the first, second, and third tests, respectively. It was consistently higher after a positive test than after a negative test (first test, 1.68 vs 0.92 per 100 person-years; second test, 1.59 vs 0.77 per 100 person-years; and third test, 1.28 vs 0.68 per 100 person-years). The proportion of individuals who had a chlamydia test on the same date as their PID diagnosis was higher in the positive than in the negative cohort (first test, 1.18% [47 of 3991] in the positive vs 0.58% [401 of 68 892] in the negative cohort; second test, 1.56% [42 of 2692] and 0.51% [286 of 55 781], respectively; and third test, 1.21% [24 of 1988] vs 0.51% [225 of 43 936]). This can be seen in the Kaplan–Meier plots of time to PID after a chlamydia test (Figure 2). The divergence between the 2 curves (positive and negative cohorts) continues and increases over the duration of follow-up.

A single positive chlamydia test increased the risk of PID during follow-up by 55% (adjusted hazard ratio [AHR], 1.55; 95% CI, 1.43–1.70) after the first test (Table 1). One previous positive test increased the risk of PID by 17% (AHR, 1.17; 95% CI, 1.06–1.30) after the second test). Two previous positive tests increased the risk by 35% (AHR, 1.35; 95% CI, 1.04–1.75). The risk of PID decreased with age and increased with subsequent testing episodes (Table 1). The highest risk of PID was seen in individuals who were younger than 16 years at the time of their third chlamydia test compared with women older than 30 years (n = 3753; AHR, 4.55; 95% CI, 3.59–5.78). There was also evidence of a time trend in the risk of PID. Individuals tested in more recent years (first test in 2005–2008) had a lower risk of PID than those tested at the start of the cohort (first test in 1992–1996; data not presented) (AHR, 0.45; 95% CI, .35–.56).

**Table 1.** Crude and Adjusted Hazard Ratios for the Risk of Pelvic Inflammatory Disease After the First, Second, and Third Chlamydia Tests

Test Results and Age	Crude or Adjusted Hazard Ratio (95% Confidence Interval)					
	1st Test		2nd Test		3rd Test	
	Crude	Adjusted^a^	Crude	Adjusted^a^	Crude	Adjusted^a^
Current test^b^						
Positive	1.85 (1.70–2.01)	1.55 (1.43–1.70)	2.07 (1.86–2.29)	1.55 (1.39–1.72)	1.89 (1.66–2.14)	1.29 (1.13–1.47)
1 previous test^c^						
Positive	…	…	1.68 (1.52–1.86)	1.17 (1.06–1.30)	…	…
2 previous tests^c^						
Negative/positive	…	…	…	…	1.81 (1.58–2.07)	1.23 (1.08–1.42)
Positive/negative	…	…	…	…	1.66 (1.47–1.87)	1.13 (1.00–1.28)
Positive/positive	…	…	…	…	2.19 (1.70–2.85)	1.35 (1.04–1.75)
Age at test, y^d^						
25–29	0.96 (.76–1.23)	0.74 (.58–.95)	1.45 (1.19–1.77)	1.04 (.85–1.27)	1.69 (1.40–2.05)	1.19 (.98–1.45)
20–24	1.07 (.85–1.35)	0.72 (.57–.91)	2.02 (1.68–2.42)	1.22 (1.01–1.48)	2.57 (2.15–3.08)	1.52 (1.26–1.83)
16–19	1.48 (1.18–1.86)	0.96 (.76–1.22)	3.61 (3.01–4.32)	1.95 (1.60–2.36)	5.31 (4.44–6.34)	2.65 (2.18–3.21)
12–15	2.63 (2.08–3.32)	1.55 (1.22–1.98)	7.06 (5.78–8.63)	3.25 (2.62–4.03)	10.79 (8.66–13.43)	4.55 (3.59–5.78)

^a^ Adjusted for age at test, year of test, region of residence, and previous test result(s).

^b^ Baseline is risk after a negative test.

^c^ Baseline risk is only previous negative test(s).

^d^ Baseline risk is age 30–40 years.

## DISCUSSION

For women who participated with an established chlamydia control program, a diagnosis of chlamydia increased their risk of PID during their reproductive lifetime by 50%, and each repeated infection increased this risk by a further 20%. The highest risk of PID was seen in individuals who were younger than 16 years at the time of their chlamydia test. These findings support targeting health promotion interventions at young women under 16 years and those with a history of chlamydia. It may also be appropriate to represent this heterogeneity in risk within transmission dynamic models of chlamydia to improve the accuracy of the predicted impact and cost-effectiveness of control interventions.

To our knowledge, this is the largest published population-based cohort of chlamydia and PID to date. We used high-quality administrative health data sets from the provincial government of Manitoba to generate a cohort that is a very close approximation to the resident population and has a high ascertainment of all public healthcare encounters for PID, ectopic pregnancy and infertility [25]. Owing to the high level of participation with a well-established chlamydia control program we were able to report the risk of PID after repeated chlamydia infections. We were able to include PID diagnoses made in primary care as well as those from a hospital setting (excluding the emergency and urgent care departments), addressing a key limitation of earlier studies [17, 18].

The advantage of a retrospective cohort is that it can provide timely findings with limited resource requirements. However, with this study design, we could not demonstrate a causal relationship between chlamydia and PID. Furthermore, because we used data that had already been collected, our findings are dependent on the quality of the information in the administrative data sets. Before 2007 a small, but unknown, number of chlamydia tests are missing from the CPL data set because some tests were performed in other settings (particularly for residents in the Rural North). This may have led to an incorrect classification of exposure status, or it may have resulted in tested persons being excluded from the cohort.

Although the MH data sets are complete, timely, and consistent (except for the change from ICD-9 to ICD-10 in the Hospital Separations Abstracts data set) [25, 26], diagnoses of PID recorded in administrative data can have a low positive predictive value for a corresponding case of PID in the medical notes [27, 28]. There is also uncertainty around the accuracy of a clinical diagnosis of PID because there is no noninvasive gold standard diagnostic test and there is a reasonable possibility that physicians may be biased toward the diagnosis in women with certain risk factors, including a previous history of chlamydia. Despite including primary care diagnoses, we may have underascertainment of PID because we were not able to include diagnoses from urgent or emergency care departments in the hospital. We also made a trade-off between completeness and accuracy in our definition of PID in primary care (because coding is limited to the first 3 digits of ICD-9 we excluded 616.0—cervicitis and endocervicitis—because 616.1–616.9 do not define PID).

We used the chlamydia tests in the CPL data set to assign chlamydia exposure status. Chlamydia tests measure exposure to chlamydia at an instant with an accuracy that depends on the test's sensitivity and specificity. We will have incorrectly assigned persons to the chlamydia-negative cohort if their infections went undetected either through a false-negative result or the absence of a test. We limited our definition of a chlamydia test to one occurring ≥60 days after a previous test to avoid the risk of false-positive results after treatment and to be consistent with an earlier study [17], but this may have led to underascertainment of new infections within 60 days of treatment. It is not possible to estimate the combined scale of this underascertainment. In addition, we made the assumption that all cases of PID were caused by chlamydia if the woman's index test was positive [18]. This is unlikely to be true. Although we were able to adjust our analysis for several potential confounding factors (age at test; year of test and region of residence) in this analysis we did not have any information about gonorrhea or sexual behavior.

Manitoba has had a well-established population-wide chlamydia control program predating the start of this cohort. Therefore, we assume that women who had chlamydia diagnosed were promptly and appropriately treated, preventing the ascension of chlamydia into the upper genital tract. Despite this, the overall rate of PID in women with a diagnosis of chlamydia was twice as high as that in women with negative tests, and there was a 50% increase in the risk of PID in those with chlamydia-positive test results (AHR, 1.55; 95% CI, 1.43–1.70). These findings can be partly explained by infection ascending in the interval between testing and treatment [29], concurrent diagnosis of chlamydia and PID and treatment failure or noncompliance. But the combined effect of these factors is unlikely to explain the magnitude of the observed increase in risk after a positive test. We suggest that the index chlamydia infection may be a marker for an unmeasured PID risk, such as repeated (undiagnosed) chlamydia or gonorrhea infections. Further research to identify the factors contributing to this increased risk in treated individuals may help structure successful control interventions.

Two earlier retrospective population-based cohorts from Uppsala, Sweden [18]. and Sør-Trøndelag County, Norway [17], have demonstrated the association between chlamydia and a hospital presentation (inpatient and outpatient) with PID. The cumulative incidence of PID in Manitoba (over a similar duration of follow-up) was much higher than those reported from Sweden or Norway in both women who tested positive for chlamydia and those who tested negative (Manitoba, 14.8% in positive and 7.8% in negative cohorts; Uppsala, 5.6% [95% CI, 4.7–6.7] and 4.0% [95% CI, 3.7–4.4], respectively; and Sør-Trøndelag, 1.09% [95% CI, .82–1.44] and 0.70% [95% CI, .59–.82]). We would expect to find a higher cumulative incidence of PID in this cohort because there are no private hospitals in Manitoba, we used a broader definition of PID, and we included diagnoses made in primary care. However, there may also be unmeasured differences between the settings that could contribute to this finding, for example, the incidence or prevalence of other STIs, the clinical definition of a case of PID, and the coding of PID in administrative data sets.

Despite this difference in cumulative incidence, the AHR of PID in women who tested positive compared with those who tested negative in Manitoba (AHR, 1.55; 95% CI, 1.43–1.70) was similar to those reported from Sweden (AHR, 1.27; 95% CI, 1.04–1.55) [18] and Norway (1.69; 95% CI, 1.21–2.37) [17]. This comparison is difficult to interpret because the analyses were adjusted for different confounders. However, the findings from all 3 studies provide good evidence that women with a diagnosed and treated chlamydia infection remain at increased risk of PID throughout their reproductive lifetime.

We found evidence to suggest that repeated infections increased the lifetime risk of PID. This is consistent with the only published study that has analyzed this association in women from the general population [19]. However, the risk of PID in women with 2 chlamydia infections was higher in the Wisconsin study than we found in Manitoba (crude odds ratio for Wisconsin study, 4.0 [95% CI, 1.6–9.9]; crude hazard ratio for Manitoba study, 2.19 [95% CI, 1.70–2.85]). Possible explanations for this are that we looked at the risk after consecutive positive tests rather than the total number of positive tests and that there may be differences between the 2 populations (for example the Wisconsin study tested women between 1985 and 1992 using tests with a lower sensitivity than the NAATs introduced in Manitoba in 1998). Our findings suggest that health promotion and screening interventions may be more beneficially targeted at women with a current or previous infection, but we are unable to comment on the mechanism for this increased risk [12].

We also demonstrated that tests performed after the introduction of NAATs carried a lower risk of progression to PID. This may be a direct result of detecting infections with lower potential to progress to PID, detecting and treating a higher proportion of infections, or it may reflect other secular changes, for example, in the risk profile of those who participate with testing or circulating gonorrhea rates. However, this is an interesting finding that warrants further research.

## CONCLUSION

There is evidence of heterogeneity in the risk of PID after chlamydia in this large population-based cohort. Young women and those with repeated infections have the highest risk and may benefit from more intensive health promotion interventions. Furthermore, describing progression to PID as an average rate, as seen in the majority of published mathematical models of chlamydia, may be an inappropriate oversimplification. Sensitivity analyses should be used to explore the impact of individual-based risks on the predicted impact of screening strategies.

## Supplementary Data

Supplementary materials are available at *The Journal of Infectious Diseases* online (http://jid.oxfordjournals.org/). Supplementary materials consist of data provided by the author that are published to benefit the reader. The posted materials are not copyedited. The contents of all supplementary data are the sole responsibility of the authors. Questions or messages regarding errors should be addressed to the author.
